# How do surgical pathologists evaluate critical diagnoses (critical values)?

**DOI:** 10.1186/1746-1596-3-30

**Published:** 2008-07-12

**Authors:** Masoud Mireskandari

**Affiliations:** 1 Pathology Department, Iran University of Medical Sciences, Tehran, Iran

## Abstract

**Background:**

After introduction of the concept of critical value (CV) in laboratory medicine, some efforts were performed to define possible critical values in surgical pathology. Critical diagnosis (critical value) is a concept recently established in surgical pathology and is a challenging issue among pathologists and clinical specialists. The concept may be the subject of variation according to the geographical or work setting differences. The current study was performed to bring the contribution of the Iranian pathologists to the evolving concept of critical diagnoses (critical values) in surgical pathology.

**Materials and methods:**

During annual meeting of Iranian Pathologist Society, November 2006, Tehran, Iran, anonymous questionnaires were distributed among participants. They were requested to openly name conditions in which a pathologist should communicate the results immediately with clinicians.

**Results:**

147 pathologists completed the questionnaire. They were varied in their level of experience and setting of workplace. Each participant referred to 1–7 (mean 3) conditions as CV. About 90 different conditions which were considered as CV by participants were extracted from the questionnaires.

**Discussion:**

The list of conditions obtained through this survey as CVs in surgical pathology covered most items previously described in literature. Major differences are low number (or lack) of refers to some relatively routine and potentially important conditions and considering many unimportant conditions as CV by participants of present survey. Almost all conducted surveys have been performed on this issue so far (including the present survey) suffer from lack of supportive scientific evidences and based mainly on experience and common sense of participants in survey. Potential problems with application of CV concept in daily routine work flow of pathology, particularly in developing countries like Iran, were discussed.

## Background

The concept of critical value (CV) was introduced for first time in 1972 by Lundberg [1]. He defined the situation as "a pathophysiologic derangement at such variance with normal as to be life threatening if therapy is not instituted immediately". This concept is very understandable in clinical laboratory settings when physicians are working with "numerical" data. Accordingly very clear cutoffs can be defined which are discriminative between life threatening emergency situations and those that can be handled in the process of routine practice. One of the best known examples of CVs in clinical laboratory is critically low levels of serum potassium which threats life by imposing patient to danger of cardiac arrhythmias and hence mandates immediate intervention. The question emerged thereafter about the possible presence of similar conditions in surgical pathology. As a discipline integrated in the field of laboratory diagnosis the presence of CVs in surgical pathology is expectable. But in contrast to clinical laboratory, surgical pathologists are engaged with "interpretation" rather than "number". In most instances surgical pathologists rely on their experience and common sense to consider a pathologic process as a CV. For considering a condition as CV in surgical pathology one important characteristic should be taken into account: it is potentially life threatening if any delay happens in diagnosis and communicating the results with clinicians. At least in developing or underdeveloped countries implementation of CV concept in practice needs much investment in equipment and dramatic changes in specimen handling. Accordingly at first step it is necessary to realize how pathologists conceptualize the CV in surgical pathology and which conditions they find necessary to immediate interaction. In this study a survey was conducted to assess the viewpoint of Iranian pathologists about the CVs in surgical pathology and to find out how close or far their ideas from their peers in developed countries, using ADASP guidelines as a base for comparison.

## Methods

During annual meeting of members of Iranian Pathologists Society, November 2006, Tehran, Iran, anonymous questionnaires were distributed among participants. In addition to general and demographic data, the participants were requested to openly notify five situations in which they think it is necessary to inform clinicians immediately about the results of surgical pathology specimens' evaluation without any delay for completion of routine process. Exact phrases in questionnaire are " Please name five clinical situation, pathologic diagnoses, or specific findings in microscopic or macroscopic evaluation of pathology specimens that when you encountered, you find it necessary to call the attending physician and communicate the findings immediately, before waiting for completion of routine process of pathology report preparation. On the other word which situations in surgical pathology seems to you as critical situations." The answers were extracted from questionnaires, tabulated and compared with available previous studies.

## Results

147 pathologists filled the questionnaire. They included 81 males and 60 females with age ranging 25–67 (mean 39.5) years. In 6 questionnaires the sex of participant was not identified. Participants are various in the level of education and experience and their workplace setting. Three of the participants were pathology assistants, all were in year 4 of postgraduate study. 41 participants were members of scientific boards of universities (38 assistant and 1 associated Professor) with 1–24 (mean 8.5) years of experience. Two scientific board members did not identify their scientific grade. 91 participants were not member of scientific board with 1–30 (mean 6.9) years of experience. Most participants were working only in university hospitals (39), followed by non-university hospitals (26) and private outpatient laboratory (24). 45 participants were working in two settings at a same time. The frequent combinations were university hospital and private outpatient lab (18), non-university hospital and private outpatient laboratory (25) and university and non-university hospital laboratories (2). In 11 questionnaires the workplace setting were not identified.

In addition to 3 assistants, 27 of participants were only responsible for clinical laboratory at the time they responded the questionnaire and were not signing out any surgical pathology specimen in their workplace. Since pathology assistants study in both fields of surgical and clinical pathology in Iran, the answers of these participants were taken into account, too. Among the other participants the number of cases each pathologist were responsible for directly, varied from 20 to 6000 in each year (mean 1336).

In each questionnaire from 1 to 7 (mean 3) conditions were notified by participants as CVs. About 90 different conditions were named by participants as critical values in surgical pathology (table 1). The number of times each condition was referred was ranged from 1 to 52. The most frequently referred situation was acute leukemia in bone marrow sampling (52) followed in decreasing order by reports of intraoperative consultation (26), unexpected malignancy in any specimens (23), and mucor mycosis (12). High grade lymphomas and insufficient or absent specimen were referred 9 times. Breast malignancies (with no further qualification) were referred as a critical value 7 times. Malignancy in endoscopic biopsy specimens, malignancy in fine needle aspiration of tissues, and malignant melanoma were referred 6 times. Among the conditions which were referred more than one time but less than 6 times, noticeable conditions include malignant cells in cytology of fluids (5), unexpected benignancy (5), any difference between interpretation of present evaluation and previous pathologic diagnosis (4), necrotizing vasculitis (4), renal needle biopsy in transplanted kidney (3), mediastinal lymphoblastic lymphoma (3), septic arthritis (3), any mediastinal mass in patients with superior vena cava syndrome (3), Burkitt's lymphoma (2), diagnostic curettage in suspected ectopic pregnancy cases (2), margins involvement in resection specimens of malignant tumors (2), rapidly progressive glomerulonephritis (2), and infectious agents in immunocompromised patients (2). About 45 conditions were referred only once by participants. Some noticeable conditions among them are: diagnostic curettage in patients suspected to malignancy, discordance between clinical and pathologic diagnosis, false sampling in confirming biopsies (tubal ligation, vagotomy, vasectomy, etc), temporal arteritis, and transplant rejection.

**Table 1:** Most common conditions considered as CV by participants of present survey.

**Conditions**	**Number of Refers**
Acute leukemia in bone marrow specimens	52
FS reports	26
Unexpected Malignancy	23
Mucor mycosis	12
All malignancies	9
High grade lymphomas	9
Unsuitable or absent specimen	9
Breast malignancy	7
Malignancy in endoscopic specimens	6
Malignancy in FNA	6
Malignant melanoma	6
Brain Tumors	5
CIN II and III	5
Malignant cells in cytology of fluids	5
Tuberculosis	5
Unexpected Benignancy	5
Bone marrow biopsy for thrombocytopenia	4
Difference with previous pathologic diagnosis	4
Necrotizing vasculitis	4
Renal needle biopsy in transplanted kidney	4
CSF cytology with malignant or leukemic cells	3
Cutaneous vesiculo-bullous diseases	3
Fluid cytology	3
Mediastinal lymphoblastic lymphoma	3
Metastatic tumors	3
Septic arthritis	3
Wilm's tumor	3
Mediastinal mass in SVC syndrome	3

Second column numbers depict how many times the condition was referred by participants.

## Discussion

"Critical value" is a well known concept in clinical pathology laboratory. It has been defined as quantitative levels of analytes in any body fluids (particularly in serum) which impose patients directly or indirectly to life threatening consequences, and hence needs rapid communication with physician and immediate intervention. The most usual examples in daily practice are very high or very low levels of serum potassium.

In surgical pathology the situation is very different from clinical laboratory setting. Surgical pathology reports' contents are usually in the form of "interpretations". Very rarely "numerical data" can be found in surgical pathology report and if they can, most often are indicators of grading or staging of a benign inflammatory (viral hepatitis) or malignant neoplastic process. Hence critical values in surgical pathology cannot be defined by "cutoffs". In addition most critical values in surgical pathology are those which depend on the clinical variables and condition of patient. For instance absence of chorionic villi, trophoblastic cells and embryonal tissue in an endometrial curettage can be considered critical only when the patient was suspected to be pregnant. Accordingly the more appropriate term might be "critical diagnoses" rather than "critical values". Such limitations hampered precise definition of CV in surgical pathology. On the other hand if in a clinical situation the problem of time plays a critical role, one of routine approaches is intraoperative consultation and frozen section examination. One can postulate that CVs in surgical pathology are those conditions which cannot be managed by frozen section examination. These are among the reasons why defining CV in surgical pathology is not straightforward and why the pathology literature including pathology textbooks are very poor on this issue.

One of the rare studies performed to define CVs in surgical pathology is a survey performed by Pereira and colleagues [2]. According to their personal experience they provided a list of 11 conditions which considered being critical values in surgical pathology. Then they presented the list to a group of 11 pathologists and 5 clinical specialists and asked them to grade the situations as a CV in a four tier scale according to the level of urgency for communication with clinicians. The situations included crescents in kidney biopsy specimen, vasculitis, bacteria in heart valve or bone marrow, organisms in an immunocompromised patient, fat in an endometrial curettage specimen, uterine contents without villi or trophoblasts in the workup of a patient suspected to be pregnant, mesothelial cells in a heart biopsy specimen, transplant rejection, malignancy in superior vena cava syndrome, neoplasms potentially causing paralysis, and large vessels in a core biopsy specimen. Additional situations were added by participants to the above list. Participating pathologists added unexpected malignancy, disagreement between frozen section and permanent diagnoses, all fine-needle aspirations performed by a pathologist, fat in snare of biopsies of a colon polyp, polyomavirus in urine cytologic specimen, hydatidiform mole, hemophagocytic syndrome, necrotizing fasciitis, staphylococcal scalded skin syndrome, and various hematologic malignant neoplasms such as acute leukemia (also listed specifically as acute myelogenous leukemia, French-American-British type M3), Burkitt lymphoma, and leukemia cutis. The additional diagnoses listed by clinicians were unexpected malignancy, change of diagnosis in inflammatory bowel disease (from Crohn disease to ulcerative colitis or vice versa), acid-fast bacilli in a tissue biopsy specimen (eg, lymph nodes), and invasive aspergillosis or fungi in the nasal sinus or lung.

The same group of scientists later conducted another survey for evaluation of critical values in cytology [3]. Critical value conditions which presented for scaling to participants were unexpected malignancy, disagreement between preliminary and final fine-needle aspiration diagnoses, and organisms in nongynecologic and fine-needle aspiration specimens. Additional CV cases suggested by the survey participants included herpes in a Pap smear in a pregnant patient, atypical glandular cells of uncertain significance in Pap smears, amended reports, very unusual tumors, disagreement with outside slide interpretation, infection or malignancy in orbital fine-needle aspiration samples, discrepancy between clinical expression and pathologic interpretation, and delay in signing out the cytology report.

Considering the long list of conditions mentioned by participants in the present survey (about 90 conditions), it is not surprising that many of conditions which has been discussed in two surveys of Pereira et al were mentioned by participants of present survey, too. But there are some concerns about the viewpoint of present survey participants. The first is the large number of refers to conditions which they are not included in the Pereira studies and rationally cannot be considered as a CV. Such conditions include breast malignancy (9), malignancy in all endoscopic specimens (6), malignant melanoma (6), brain tumors (6), and CIN II and III (5). The second is very low number of refers to the conditions which are considered as CV in the Pereira studies. Such conditions include diagnostic curettage in suspected ectopic pregnancy (2) and presence of crescents in kidney biopsy specimens (2). There are some important conditions in the Pereira studies which were never mentioned by participants in present survey. These include bacteria in heart valve or bone marrow, organisms in an immunocompromised patient, fat in an endometrial curettage specimen, mesothelial cells in a heart biopsy specimen, large vessels in a core biopsy specimen, fat in snare of biopsies of a colon polyp, and acid-fast bacilli in a tissue biopsy specimen. Although many of these conditions are rare in general pathology laboratories and are only encountered in specific situations (e.g. mesothelial cells in heart muscle biopsy), many of them are among the conditions that may happen in every general hospital pathology laboratory (e.g. fat in endometrial curettage, fat in the snare of biopsies of colonic polyps, or large vessels in needle biopsy specimens). This comparison shows that the general knowledge about the critical values in surgical pathology is poor among pathologists community. One of the reasons may be the rarity of documents covering this important issue in pathology literature. Dedication of a book chapter or appendices of textbooks to this topic can definitely attract attention of under or post graduate pathologist to this important topic.

To fill this gap in our knowledge, in 2006 the Association of Directors of Anatomical and Surgical Pathology (ADASP) provided a list of CVs in surgical pathology [4-6]. Based on the survey among ADASP members, the following conditions were extracted and categorized under three different headings. Group one is conditions that have immediate clinical consequences. This group includes crescents in greater than 50% of glomeruli in a kidney biopsy, leukocytoclastic vasculitis, uterine contents without villi or trophoblast, fat in an endometrial curettage, mesothelial cells in a heart biopsy, fat in colonic endoscopic polypectomies, transplant rejection, malignancy in superior vena cava syndrome, and neoplasms causing paralysis. The second group is composed of conditions in which there are unexpected or discrepant findings. This group includes significant disagreement between frozen section and final diagnosis, significant disagreement between immediate interpretation and final FNA diagnosis, unexpected malignancy, and significant disagreement and/or change between primary pathologist and outside pathologist consultation (at either the original or consulting institution). The third group is constituted by infectious conditions. They include bacteria or fungi in CSF cytology in immunocompromised or immunocompetent patients, pneumocystis, fungi or viral cytopathic changes in bronchoalveolar lavage (BAL), bronchial washing or brushing cytology specimens in immunocompromised or immunocompetent patients, acid-fast bacilli in immunocompromised or immunocompetent patients, fungi in FNA of immunocompromised patients, bacteria in heart valve or bone marrow, herpes in Pap smears of near term pregnant patients, and any invasive organism in surgical pathology specimens of immunocompromised patients. ADASP committee has emphasized that the above list would be considered only as a template and each institute should define their list individually and by cooperation with clinical colleagues. They also commented to avoid overuse of this terminology.

Preparation of the template table by ADASP members is a step forward in the issue of critical values in surgical pathology, but it seems insufficient. Many questions and problems remained unsolved in this issue and deserve more attention.

▪ **Are pathologists responsible for defining critical values in surgical pathology? **It seems clear that "critical value" concept belongs to the pathologist and is their concern. But are they eligible to define CVs? As ADASP committee recommended any definition of CVs in surgical pathology should be discussed with clinicians. As the main "client" of pathology services clinicians have the right to define their expectations and clarify conditions they are in need of immediate communication with pathologists for the sake of better patients' management. From this viewpoint clinical specialty societies have a greater responsibility in defining CVs in surgical pathology.

▪ **Are defined CV conditions in surgical pathology supported by scientific evidence? **As a matter of fact most participating scientists in the Pereira and ADASP surveys considered a condition as a CV in surgical pathology, largely according to their experience. It seems that none of surveys tried to critically search for supportive or non-supportive documents. Accordingly almost all the listed conditions in the ADASP recommendation could be potentially a subject of research. Studies should be conducted by pathologists to clarify much vagueness in CV concept in surgical pathology. As described before, the key feature of a CV in surgical pathology is that any delay in diagnosis or communicating results with clinicians has grave consequences for patients. For each CV in surgical pathology well designed studies should verify such a potential threat in an "evidence based" approach.

▪ **How the concept of "critical values" in surgical pathology should change the pathology laboratories workflow? **Is it sufficient for pathologists to only communicate the results rapidly with clinicians? For instance if we accept that every endometrial curettage sampling can potentially be accompanied by threat of uterine wall piercing and hence presence of fat in these samples might be the first clue to this life threatening complication, any delay in processing and reporting may adversely affect patient's safety. It seems unwise to process the tissue in ordinary timetable of pathology laboratory, review the prepared slide 24–48 hours later, spend another 24 hours for submission of remaining tissue or preparation of re-cuts and then report the presence of fat in curettage content 48–72 hours after submission of specimen. It is clear that rapid communication with the surgeon after this vital gap has little, if any, effect on lowering patients' morbidity or mortality. In such situations complete submission of tissue immediately after receiving, fixing and processing the tissue by rapid processing systems, and preparation of multiple cuts are necessary changes of policy that surgical pathology laboratories' directors should consider. In fact in developed countries many of such changes has been done before. Achievements in technology of tissue processing and advent of automatic routine, specific chemical and Immunohistochemical staining procedures along with their application in routine practice have reduced turnaround time within pathology laboratories. Centralization helps in reducing costs. Communication facilities, appropriate programming, and sophisticated hospital and laboratory information systems, make immediate communication of pathology results in CV conditions very easy. Usually it is accomplished by few key strokes and minor modifications in information system. During recent years application of digital imaging in routine surgical pathology diagnosis and access to telepathology services have increased the speed and accuracy of diagnosis [7]. But what is the situation in developing countries like Iran? In contrast with developed countries specimen handling and processing methods are traditional and communication systems including laboratory and hospital information systems are not ideal. Almost always the results are not accessible by physicians before it is printed on paper and patient or his/her relatives deliver it to the physician. In such condition the only way of rapid communication is calling physician. Most pathology laboratories are small and medium sized with annual case numbers rarely reach 4000–6000. Lack of centralization and poor resources makes investment in modern facilities very difficult for most laboratories. Although previous studies showed that application of telepathology by still images can help in more accurate diagnosis, it had little effect in reducing turnaround time [8]. Hence sufficient supportive evidences and thorough cost-effect analyses are needed, before any changes in strategy are seriously considered. Every change would be rationalized before. This magnifies the importance of evidence based approach to the issue of CV in surgical pathology.
